# Radiomic Deformation and Textural Heterogeneity (R-DepTH) Descriptor to Characterize Tumor Field Effect: Application to Survival Prediction in Glioblastoma

**DOI:** 10.1109/TMI.2022.3148780

**Published:** 2022-06-30

**Authors:** Marwa Ismail, Prateek Prasanna, Kaustav Bera, Volodymyr Statsevych, Virginia Hill, Gagandeep Singh, Sasan Partovi, Niha Beig, Sean McGarry, Peter Laviolette, Manmeet Ahluwalia, Anant Madabhushi, Pallavi Tiwari

**Affiliations:** Biomedical Engineering Department, Case Western Reserve University, Cleveland, OH 44106 USA; Department of Biomedical Informatics, Stony Brook University, Stony Brook, NY 11794 USA; Biomedical Engineering Department, Case Western Reserve University, Cleveland, OH 44106 USA; Imaging Institute, Cleveland Clinic, Cleveland, OH 44106 USA; Department of Radiology, Northwestern University, Evanston, IL 60208 USA; Radiology Department, Newark Beth Israel Medical Center, Newark, NJ 07112 USA; Interventional Radiology Department, Cleveland Clinic, Cleveland, OH 44106 USA; Biomedical Engineering Department, Case Western Reserve University, Cleveland, OH 44106 USA; Radiology Department, Medical College of Wisconsin, Milwaukee, WI 53226 USA; Radiology Department, Medical College of Wisconsin, Milwaukee, WI 53226 USA; Brain Tumor and Neurooncology Center, Cleveland Clinic, Cleveland, OH 44106 USA; Biomedical Engineering Department, Case Western Reserve University, Cleveland, OH 44106 USA; Biomedical Engineering Department, Case Western Reserve University, Cleveland, OH 44106 USA

**Keywords:** Glioblastoma, survival, field-effect, biomechanical deformations, LASSO

## Abstract

The concept of tumor field effect implies that cancer is a systemic disease with its impact way beyond the visible tumor confines. For instance, in Glioblastoma (GBM), an aggressive brain tumor, the increase in intracranial pressure due to tumor burden often leads to brain herniation and poor outcomes. Our work is based on the rationale that highly aggressive tumors tend to grow uncontrollably, leading to pronounced biomechanical tissue deformations in the normal parenchyma, which when combined with local morphological differences in the tumor confines on MRI scans, will comprehensively capture tumor field effect. Specifically, we present an integrated MRI-based descriptor, radiomic-Deformation and Textural Heterogeneity (r-DepTH). This descriptor comprises measurements of the subtle perturbations in tissue deformations throughout the surrounding normal parenchyma due to mass effect. This involves non-rigidly aligning the patients’ MRI scans to a healthy atlas via diffeomorphic registration. The resulting inverse mapping is used to obtain the deformation field magnitudes in the normal parenchyma. These measurements are then combined with a 3D texture descriptor, Co-occurrence of Local Anisotropic Gradient Orientations (COLLAGE), which captures the morphological heterogeneity and infiltration within the tumor confines, on MRI scans. In this work, we extensively evaluated r-DepTH for survival risk-stratification on a total of 207 GBM cases from 3 different cohorts (Cohort 1 (*n*1 = 53), Cohort 2 (*n*2 = 75), and Cohort 3 (*n*3 = 79)), where each of these three cohorts was used as a training set for our model separately, and the other two cohorts were used for testing, independently, for each training experiment. When employing Cohort 1 for training, r-DepTH yielded Concordance indices (C-indices) of 0.7 and 0.65, hazard ratios (HR) and Confidence Intervals (CI) of 10 (6 – 19) and 5 (3 – 8) on Cohorts 2 and 3, respectively. Similarly, training on Cohort 2 yielded C-indices of 0.6 and 0.7, HR and CI of 1 (0.7 – 2) and 3 (2 – 5) on Cohorts 1 and 3, respectively. Finally, training on Cohort 3 yielded C-indices of 0.75 and 0.63, HR and CI of 24 (10 – 57) and 12 (6 – 21) on Cohorts 1 and 2, respectively. Our results show that r-DepTH descriptor may serve as a comprehensive and a robust MRI-based prognostic marker of disease aggressiveness and survival in solid tumors.

## I. Introduction

It is well recognized that cancer is not a bounded, self-organized system [1], but a systemic disease. Most malignant tumors have heterogeneous growth patterns, leading to disorderly proliferation well beyond the visible surgical margins. In fact, in solid tumors, depending on the malignant phenotype, the impact of the tumor is observed not just within the visible tumor, but also in the immediate peri-tumoral, as well as in seemingly normal-appearing adjacent regions [1], [2], a phenomenon known as ‘tumor field effect’ [3]. For instance, Glioblastoma (GBM), one of the most aggressive brain tumors, is known to extend several millimeters distal to the tumor margins, which ultimately leads to recurrence in GBM patients [4]. Similarly, the herniation or gross distortion of the brainstem, remote to the tumor location, is the proximal cause of deaths in 61% of GBM studies [5]. The infiltrating brain tumor mass pushes and displaces the surrounding tissue structures (known as mass effect), leading to a mid-line shift and an increase in the intracranial pressure [6], [7], which ultimately results in destroying white matter tracts and alterations of consciousness and other chronic conditions such as seizures and headaches in GBM patients. While tumor infiltration [8], [9] and mass effect [10], [11] have both, to different extents, been shown to be associated with more aggressive tumor behavior and poor prognosis, it may be reasonable to assume that there might be latent disease-specific information to quantify both phenomena on routine imaging. Specifically, mass effect may be captured via the subtle tissue deformations in the seemingly-normal brain regions adjacent to tumor (also known as “brain around tumor (BAT)”). The rationale being that more aggressive tumors may exert increased intra-cranial pressure on the surrounding BAT regions, resulting in pronounced structural deformations and thus worse prognosis, as compared to less aggressive tumors. Additionally, the pronounced deformations, when combined with features from within the tumor confines that may quantify intra-tumoral heterogeneity and tumor infiltration, may serve as image-based prognostic markers of overall survival in GBM tumors.

“Recently, ‘radiomics’ (i.e. extraction of quantitative image features such as co-occurrence, gray-level dependence, directional gradients, and shape-based) has provided a surrogate mechanism to non-invasively capture sub-visual cues of intra-tumoral morphological heterogeneity on routine imaging, for different prognostic and predictive applications. Many studies in the literature have involved machine and deep-learning approaches using routine magnetic resonance imaging (MRI) sequences and have shown potential in survival prediction and response assessment for brain tumors [12]–[20]. Specifically, the recent works in [19], [20] attempted to segment the tumor sub-compartments using pre-operative scans, then extracted radiomic features from these sub-compartments and combined them with clinical information such as age and resection status, into machine learning algorithms to predict patient overall survival. These studies have largely focused on capturing local textural changes within the tumor and the peri-tumoral regions [21], and their associations with patient survival [22]. However, a missing gap in previous work has been to leverage the subtle tumor-induced deformations in the BAT regions as measured on routine MRI scans, as a complementary radiomic marker for prognosticating patient survival. In this context, our group developed an integrated MRI-based descriptor, that captures radiomic-Deformation and Textural Heterogeneity (r-DepTH) [23], which accounts for both the tumor-induced deformations in the BAT regions as well as the intra-tumoral heterogeneity from within its visible confines. Overall, the r-DepTH descriptor involves capturing phenotypic attributes of tumor infiltration as well as mass effect, as both of these aspects, to varying degrees, have been shown to be associated with worse outcomes in GBM tumors. This is achieved by computing Co-occurrence of Local Anisotropic Gradient Orientations (COLLAGE) descriptor [24] from the tumor and the surrounding peritumoral regions to quantify intra-tumoral heterogeneity and tumor infiltration, as well as computing the local biomechanical deformations to quantify mass effect and its impact on the rest of the brain. This work expands on the original implementation of r-DepTH [23] in several aspects, including rigorous robustness analysis and registration strategies across different data cohorts and extensive comparisons with existing radiomic and deep-learning strategies, as well as clinical parameters, as shown throughout the paper.

This paper is organized as follows. In Section 2, previous work on characterizing field effect in survival prediction of GBM tumors using routine MRI scans is discussed. In Section 3, we describe the methodology for computation of the r-DepTH descriptor. The experimental design and implementation details for the risk assessment model are provided in Section 4, followed by results in Section 5. Section 6 provides discussion and concluding remarks.

## II. Previous Work and Novel Contributions

The concept of interrogating the tumor field using routine MRI scans has gained significant interest over the years, both to study its impact on tumor growth as well as correlating its impact on overall patient survival [25]–[27]. For instance, works have previously developed deterministic mathematical models that model cancerous growths from aggressive cellular proliferation in GBM tumors [25], [28], [29]. Through these models, studies investigated the induced significant mechanical stress on the surrounding tissue that results in mass effect in GBM tumors [25], [26]. These mathematical models consider the cellular motility factor in GBMs, to account for its invasiveness as well as the ability of its cells to migrate and proliferate [25]. A mechanically coupled model was also suggested in [27], to address the mechanical stress caused by tumor expansion, while also incorporating a diffusion coefficient that accounts for local tissue stress due to the field effect. In addition to these deterministic mathematical models, multiple studies have explored the utility of ‘data driven’ approaches, such as radiomic features extracted from GBM patients in survival prediction. For instance, [14] showed that radiomic features outperformed clinical and radiologic risk models in predicting overall survival in GBM tumors. Similarly, the studies conducted by [15], [30]–[32] assessed the utility of texture features extracted from the tumor and peritumoral regions for survival prediction in GBM. Our own group has developed a textural radiomic descriptor, COLLAGE [24], that has demonstrated success in capturing subtle differences between similar appearing disease phenotypes on routine imaging, across different types of malignant tumors [33], [34]. COLLAGE captures local anisotropic differences in intra-tumoral heterogeneity by calculating per-voxel gradient orientations, followed by obtaining statistics of Gray-Level Co-Occurrence Matrix (GLCM) heterogeneity to quantify patterns of local gradient alignment. However, those existing prognostic studies in GBM have been limited to interrogating texture representations from the enhancing lesion, inner necrotic core, and peritumoral area, and have not explicitly accounted for any possible biomechanical deformational changes in the BAT region.

**Fig. 1.**
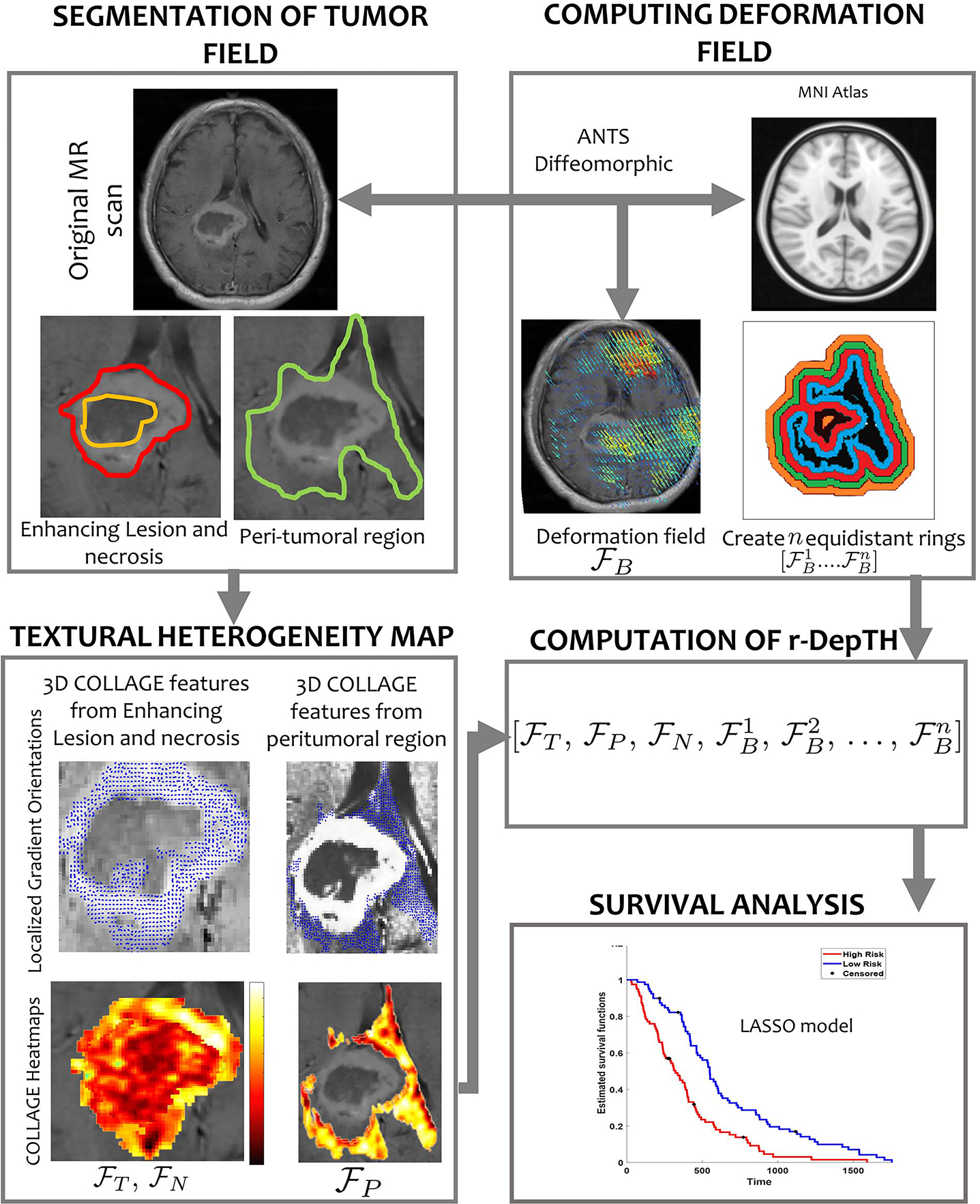
Overview of r-DepTH framework. First, segmentation of tumor compartments of interest (enhancing lesion (outlined in red), peri-tumoral area (outlined in green), and necrotic core (outlined in orange)) is performed. Following pre-processing, feature extraction is performed via COLLAGE features to characterize the intra-tumoral textural heterogeneity, and deformation heterogeneity features to characterize the tumor impact on BAT region. Next, the sets of COLLAGE and deformation features are concatenated to compute the r-DepTH descriptor. The r-DepTH descriptor could then be employed for classification/survival analysis (in our case using a LASSO model for stratifying GBM patients into low- and high-risk groups based on their overall survival).

The key contribution of this work is the development of r-DepTH descriptor that combines measurements of biomechanical and morphological features of the tumor regions, for predicting patient survival. Briefly, we capture the biomechanical deformations in the BAT regions by using diffeomorphic registration between the GBM brain scans and a healthy atlas. We then utilize the inverse mapping during registration to calculate the deformation magnitudes within uniformly sized annular sub-volumes that belong to the surrounding BAT regions, both adjacent and distal to the tumor boundaries (as close as 5*mm* and up to 60*mm*). In addition, we use 3D COLLAGE features [24] to capture the textural heterogeneity from within the tumor visible confines. The deformation features from the normal parenchyma and the COLLAGE features from the tumor confines, are finally combined to obtain the integrated r-DepTH descriptor. Figure 1 provides an overview of the r-DepTH framework.

## III. Methodology

### A. Notation

We define an image scene *I* as *I* = (*C, f*), where *I* is a spatial grid of voxels *c* ∈ *C*, in a 3-dimensional space, *R*^3^. Each voxel, *c* ∈ *C*, is associated with an intensity value *f* (*c*). *I*_*T*_, *I*_*P*_, *I*_*N*_, and *I*_*B*_ correspond to the intra-tumoral, peri-tumoral, necrotic, and surrounding normal parenchymal sub-volumes within every *I* respectively, such that [*I*_*T*_, *I*_*P*_, *I*_*N*_, *I*_*B*_] ⊂ *I*. We further divide the sub-volume *I*_*B*_ into uniformly sized annular sub-volumes $I_{B_{j}}$, where *j* is the number of uniformly-sized annular bands, such that *j* ∈ {1, …, *m*}, and *m* is a user-defined proximity parameter that is dependent on the distance from the tumor margin. We extract each feature set ℱ from each training *S*_*t*_ and test *S*_*v*_ set, across the different cohorts employed in this study. The common notations and acronyms employed in this paper are listed in Table I.

### B. r-DepTH Descriptor

#### 1) Deformation Heterogeneity Features From the Normal Parenchyma:

Healthy T1-w MNI atlas (*I*_*Atlas*_) is used to measure the tissue deformation in the normal appearing brain regions of every patient volume *I*. *I*_*Atlas*_ is first non-rigidly aligned to *I* using mutual-information-based similarity measure provided in ANTs (Advanced Normalization Tools) SyN (Symmetric Normalization) toolbox [35]. This toolbox is specifically used due to its proved efficiency in mapping brain images containing lesions into healthy templates [36]. It also efficiently handles the constrained cost-function masking approach, where the mapping within a tumor exclusive region is determined by the solution of the negative tumor mask region *I*_*mask*_. We employ Lagrangian diffeomorphic-based registration [37], as it possesses symmetry properties required for a geodesic connecting two images in the space of diffeomorphic transformation that guarantees symmetry regardless of the chosen similarity measure [35]. We also wanted to ensure that only the intensity differences due to structural deformations are accounted for, during registration, while excluding the intensity differences within the tumor area. *I*_*mask*_ was hence removed from *I* during registration to *I*_*Atlas*_. Given the reference (*I*) and floating (*I*_*Atlas*_), the non-rigid alignment can be formulated as: (*I, I*_*mask*_) = *Tr*(*I*_*Atlas*_), where *Tr*(.) is the forward transformation of the composite voxel-wise deformation field (including affine components) that maps the displacements of the voxels between the reference and floating volumes. This transformation also propagates the atlas brain mask (*I*_*atlas*_) to the subject space, thereby skull stripping the subjects. As ANTs SyN satisfies the conditions of a diffeomorphic registration, an inverse *Tr*^−1^(.) exists, that successfully maps *I* to the *I*_*Atlas*_ space. This inverse mapping yields the tissue deformation of *I* with respect to *I*_*Atlas*_, representing the deformations exerted on every *c* ∈ *C*_*B*_, due to tumor mass effect. Considering $\left(c_{t}^{\prime },c_{u}^{\prime },c_{v}^{\prime }\right)$ as new voxel positions of *I* when mapped to *I*_*Atlas*_, the displacement vector is given as [*δt, δu, δv*] where vector $\left(c_{t}^{\prime },c_{u}^{\prime },c_{v}^{\prime }\right)=\left(c_{t},c_{u},c_{v}\right)+(\delta t,\delta u,\delta v)$, and the magnitude of deformation is given by calculating the Euclidean norm of the scalar values of the deformation orientations as:

(1) $$D(c)=\sqrt{{\left(\delta t\right)}^{2}+{\left(\delta u\right)}^{2}+{\left(\delta v\right)}^{2}},$$

for every $c\in C_{B_{j}}$, and *j* ∈ {1, ….., *m*}. First order statistics (i.e. mean, median, standard deviation, skewness, and kurtosis) are then calculated by aggregating *D*(*c*) for every *c* within every sub-volume $I_{B_{j}}$ yielding a feature descriptor ${ℱ}_{B_{j}}$ for every annular sub-region $C_{B_{j}}$, where $C_{B_{j}}\subset C_{B}$, *j* ∈ {1, …, *m*}.

#### 2) 3D COLLAGE Features From Within the Tumor Confines:

COLLAGE, a 3D gradient-based texture descriptor, captures intra-tumoral heterogeneity by calculating local per-voxel gradient orientations [24]. Briefly, for every voxel *c*, intensity gradients in *X, Y, Z* directions are calculated, followed by centering a 3*D* window around every *c* to compute the vector gradient matrix *F*. Then, two principal orientations, *θ*(*c*) and *ϕ*(*c*), can be obtained from *F* for every *c*, followed by computing two separate co-occurrence matrices, *M*^*θ*^ and *M*^*ϕ*^, that capture orientation pairs between voxels in a local neighborhood. From every co-occurrence matrix, a total of 13 Haralick statistics are calculated for every *c* [38]. We finally obtain first order statistics (mean, median, standard deviation, skewness, and kurtosis) for every *c* ∈ {*C*_*P*_, *C*_*T*_, *C*_*N*_}, which yields a feature descriptor ${ℱ}_{T}$ for the enhancing lesion, ${ℱ}_{P}$ for the T2/FLAIR hyperintensities, and ${ℱ}_{N}$ for the necrotic areas. Additional details regarding COLLAGE computation can be found in [24].

#### 3) Construction of r-DepTH Descriptor:

The r-DepTH descriptor is obtained for every patient, by concatenating the deformation descriptor, ${ℱ}_{B}$, along with COLLAGE texture descriptors, ${ℱ}_{T}$, ${ℱ}_{P}$, and ${ℱ}_{N}$, as ${ℱ}_{rDepTH}=\left[{ℱ}_{B},{ℱ}_{T},{ℱ}_{P},{ℱ}_{N}\right]$. ${ℱ}_{rDepTH}$ descriptor can then be employed within a supervised or an unsupervised approach for disease characterization. The algorithm for computing r-DepTH is provided in Algorithm 1.

**Algorithm 1:** Computation of r-DepTH Descriptor

**Data**: *I*, *I_Atlas_*, ROI Volume *C*
**Result:** ${ℱ}_{rDepTH}$

**begin**
**1- Obtain Deformation Features** ${ℱ}_{B}$
**for** *each patient volume **I*** **do**
Remove *Imask* from *I*, align *I_Atlas_* to *I* to get
(*I*, *I_mask_*) = *Tr*(*I_Atlas_*)
**end**
**for** *each* *c* ∈ *C_b_* **do**
Get the deformation of *I* w.r.t. *I_Atlas_*, [*δt*, *δu*, *δv*], using *Tr*^−1^ (.).
Get deformation magnitude $D(c)=\sqrt{{\left(\delta t\right)}^{2}+{\left(\delta u\right)}^{2}+{\left(\delta v\right)}^{2}}$.
**end**
**for** *each annular sub-region* $C_{B_{j}}\subset C_{B}$ **do**
Aggregate *D*(*c*) for every *c* within sub-volume $I_{B_{j}}$.
Calculate first order statistics for $I_{B_{j}}$ to get ${ℱ}_{B_{j}}$.
**end**
**2- Obtain 3D COLLAGE Features** ${ℱ}_{T}$, ${ℱ}_{P}$
**for** *each voxel* *c* ∈ *C* **do**
Obtain gradients *∂f_i_*(*c*) along *i*-axes, $\partial f_{i}(c)=\frac{\partial f(c)}{\partial i}$, *i* ∈ *x*, *y*, *z*.
**end**
Define 𝒩×𝒩×𝒩 neighborhood centered around *c* ∈ ***C***.
**for** *each voxel* *c* ∈ *C* **do**
Calculate gradient vectors ${\vec{\partial f}}_{i}\left(c_{k}\right)$ in 𝒩×𝒩×𝒩, *i* ∈ (*x*, *y*, *z*), $k\in \{1,\ldots ,{𝒩}^{3}\}$, where
$\partial f_{i}(c)={\left[\partial f_{i}\left(c_{1}\right)\;\partial f_{i}\left(c_{2}\right)\cdot \partial f_{i}\left(c_{N^{3}}\right)\right]}^{T}$
Obtain localized gradient vector matrix
$\vec{ℱ}=\left[\vec{\partial f_{X}}\left(c_{k}\right)\;\vec{\partial f_{Y}}\left(c_{k}\right)\;\vec{\partial f_{Z}}\left(c_{k}\right)\right]$
Calculate dominant components *Ψ*(*c*_*k*11_), *Ψ*(*c*_*k*12_), and *Ψ*(*c*_*k*13_),
$k\in \left\{1,\ldots ,{𝒩}^{3}\right\}$, by singular value decomposition of $\vec{ℱ}$
Obtain dominant directions *θ*(*c_k_*) and *ϕ*(*c_k_*), using
$\theta^{3D}\left(c_{k}\right)=\tan^{-1}\frac{\psi_{Y}\left(c_{k}\right)}{\psi_{X}\left(c_{k}\right)}$ and
$\phi^{3D}\left(c_{k}\right)=\tan^{-1}\frac{\psi_{Z}\left(c_{k}\right)}{\sqrt{\psi_{Y}^{2}\left(c_{k}\right)+\psi_{X}^{2}\left(c_{k}\right)}}$
**end**
Compute co-occurrence matrices *M^θ^* and *M^ϕ^* from *θ*(*c_k_*) and *ϕ*(*c_k_*)
**for** *each voxel* *c* ∈ {*C_p_*, *C_t_*} **do**
Get 13 Haralick statistics $\left[S_{\theta_{b}},S_{\phi_{b}}\right]$, *b* ∈ [1, 13] from *M^θ^* and *M^ϕ^*
Calculate first order statistics to get ${ℱ}_{T}$, ${ℱ}_{P}$, ${ℱ}_{N}$
**end**
**3- Compute r-DepTH** ${ℱ}_{rDepTH}$
Concatenate ${ℱ}_{B}$, ${ℱ}_{T}$, ${ℱ}_{P}$, and ${ℱ}_{N}$ to get ${ℱ}_{rDepTH}$
**end**

## IV. Experimental Design

While the r-DepTH descriptor is generalizable, in this work, we calculated the ${ℱ}_{rDepTH}$ from routine pre-treatment MRI scans as a prognostic marker for overall survival in GBM subjects. Details on experimental design are provided below.

### A. Data

A total of 207 cases were collected from 3 different cohorts for this study, including the publicly available Cancer Imaging Archive (TCIA) ([39]), Cleveland Clinic (CCF), and Medical College of Wisconsin (MCW). TCIA is an open archive of radiological scans for different cancer types including GBM consisting of MRIs and its associated clinical metadata, with regulations and policies for the protection of human subjects and approvals by institutional review boards in place. Data analysis from CCF and MCW was approved by the institutional Ethics Committee. Our inclusion criteria included the availability of (1) 1.5 Tesla (T) routine MRI sequences (Gadolinium (Gd)-contrast *T*_1*w*_, *T*_2*w*_, *T*_2*w*_-FLAIR) for treatment-naive patients with diagnostic image quality, and (2) Overall Survival (OS) information. This inclusion criteria yielded a total of (1) 75 GBM studies from TCIA, (2) 53 studies from CCF, and (3) 79 studies from MCW. In our experiments, we interchangeably used one of the three cohorts for training (*S*_*t*_) and used the other two cohorts independently for testing $\left(S_{v_{1}},S_{v_{2}}\right)$. Table II details the associated patient demographics for each of the 3 cohorts.

### B. Preprocessing

A total of three experts were asked to perform the manual annotations on every MRI slice, via a hand-annotation tool in 3D Slicer. The senior-most expert (V.H, *>*10-years of experience in neuro-radiology) independently annotated the studies obtained from CCF, while expert 2 (V.S) with 7 years of experience in neuro-radiology supervised expert 3 (K.B, with 3 years of radiology experience), to manually annotate the TCIA and MCW cases. In rare cases with disagreement across the two readers (expert 2 and expert 3), the senior-most radiologist (V.H, expert 1) was consulted to obtain the final segmentations. Every tumor was annotated into three regions: enhancing lesion (*I*_*T*_), *T*_2*w*_/FLAIR hyperintense peri-lesional component (*I*_*P*_), and necrotic core (*I*_*N*_). *T*_1*w*_ MRI scans were used to delineate *I*_*T*_ and *I*_*N*_, while both *T*_2*w*_ and FLAIR scans were used to annotate *I*_*P*_. Following segmentation, for every patient study, the 3 MRI sequences, Gd-*T*_1*w*_ MRI, *T*_2*w*_, and FLAIR were co-registered to a brain atlas (MNI152; Montreal Neurological Institute) using ANTs (Advanced Normalization Tools) SyN (Symmetric Normalization) toolbox [35]. Skull stripping was performed simultaneously during registration of *I* with *I*_*Atlas*_, as detailed in Section 3.2.1. Finally, bias field correction was conducted using a non-parametric non-uniform intensity normalization technique [40].

### C. Implementation Details

We calculated the deformation features ${ℱ}_{B_{j}}$ for *j* ∈ {1, 2, …, 12} annular regions that are equidistant to each other at a distance of 5*mm*. Specifically, we created concentrated annular rings around the tumor mask boundaries, using morphological operations, by dilating the tumor mask at several distances that were 5 mm apart for a total distance of 60 mm from the tumor mask boundaries, generating a total of 12 annular rings. The choice for the size of the annular rings was based on retrospective studies that have recommended 5*mm* as safe clinical target volume margin for GBM tumors [41]. This resulted in a 60 × 1 deformation vector that included 5 statistics (mean, median, standard deviation, skewness, and kurtosis) calculated for each of the 12 bands. This resulted in 12 × 5 = 60 features corresponding to ${ℱ}_{B}$. In addition, the 5 statistics were similarly obtained for each of the 13 Haralick statistics across each of the two co-occurrence matrices, resulting in 13 × 5 × 2 = 130 COLLAGE features that are extracted from each of the three compartments;${ℱ}_{T}$, ${ℱ}_{P}$, and ${ℱ}_{N}$. The descriptor ${ℱ}_{rDepTH}$ was finally obtained by aggregating ${ℱ}_{B}$, ${ℱ}_{T}$, ${ℱ}_{P}$, and ${ℱ}_{N}$. Hence the ${ℱ}_{rDepTH}$ descriptor for every tumor included a total of 450 features.

### D. Survival Risk Assessment

Following computation of ${ℱ}_{\alpha }$, where *α* = {*T, P, N, B, rDepT H*}, feature selection (reduction) was conducted using least absolute shrinkage and selection operator (LASSO), along with a cox regression model [42] on *S*_*t*_. We used LASSO to utilize its capability in reducing variance when shrinking features, while simultaneously not increasing the bias. The top features selected by LASSO model were then used to create a continuous survival risk score (*Risc*), calculated as:

(2) $$Risc\left({ℱ}_{\alpha }\right)=\sum_{g=1}^{A}q_{g}{ℱ}_{\alpha }^{g}$$

where *A* represents the number of selected imaging features from LASSO, ${ℱ}_{\alpha }^{g}$ is the *g*th feature for *α* = {*T, P, N, B, rDepT H*} and *q*_*g*_ is the respective coefficient. An observation was deemed censored if the patient withdrew from the study or there was no follow up available. All *Risc* scores were obtained based on the threshold value provided by the fitted cox model, to stratify patients into high-risk and low-risk groups obtained from *S*_*t*_. Log-rank test along with Kaplan Meier (KM) survival analysis were then performed to see how the survival rate varies between the two identified risk groups. Additionally, performance measures such as hazard ratios (HR), 95% Confidence Interval (CI), and Concordance index (C-index) were obtained to assess the performance of our survival models. HR is defined as the risk of experiencing the event of interest at a time point [43], whereas CI measures the level of uncertainty about the point estimates [44]. C-index was calculated using R (v4.0), and is commonly used to validate the predictive ability of a survival model by calculating the probability of concordance between the predicted and the observed survival [45]. Finally, on *Sv*, the top features obtained from *S*_*t*_ were used to calculate *Risc* for every patient, followed by the log-rank test to obtain the level of significance between the two identified risk groups (low-risk and high-risk).

### E. Comparative Strategies

In order to evaluate the efficacy of r-DepTH for GBM survival prediction, we performed the following comparisons: (1) Employing clinical features (age, gender, tumor volume, molecular information), in uni-variate and multi-variate settings, (2) Evaluating $Risc\left({ℱ}_{T,P,N}\right)$ using ${ℱ}_{T}$, ${ℱ}_{P}$, and ${ℱ}_{N}$, (3) Evaluating $Risc\left({ℱ}_{B}\right)$ using ${ℱ}_{B}$, (4) State-of-the-art radiomics [46], and convolutional neural network (CNN) [16] approaches previously used in the literature for GBM prognosis, and (5) hybrid approaches where (a) age and gender information were combined with ${ℱ}_{rDepTH}$ and (b) deep features obtained via a CNN approach were combined with ${ℱ}_{rDepTH}$.

Table III details the feature families for all the comparative approaches as well as the total number of extracted features from each feature family and the number of features that were selected by the LASSO survival model for survival prognostication. When employing the comparative radiomic approach on *C*_*T*_, *C*_*P*_,*C*_*N*_ regions using the CapTk software as described in [46], this resulted in a total of 4376 features $\left({ℱ}_{Rad}\right)$ for every tumor region in *S*_*t*_. This was followed by feeding $\left({ℱ}_{Rad}\right)$ to our LASSO model to calculate $Risc\left({ℱ}_{rad}\right)$ for every patient.

Additionally, we compared the performance of the r-DepTH descriptor to a CNN model previously utilized in the literature [16], to predict survival in GBM. This model is similar to most of the models found in literature in the context of survival prediction in GBM [17], [18], where transfer learning has been exploited to stratify patients into risk groups in GBM. Specifically, we extracted deep features from the GBM patients using a pre-trained CNN model via transfer learning using the strategy presented in [16]. We extracted deep features from one single tumor image that included the largest tumor region for each subject across all cohorts. The CNN acted as a feature extractor only and the obtained deep features from the CNN were then fed into the LASSO model for risk stratification. The CNN contained 5 convolutional layers and 3 fully-connected layers. The model was trained on ImageNet database with predetermined weights that are summarized in Table III. The input to the model was the cropped tumor sub-regions from the MR scans, obtained from the slice that had the largest tumor area for every patient, followed by resizing the sub-regions to 224 × 224 with bicubic interpolation. Deep features were computed by forward propagation, and extracted from the second-last fully-connected layer (similar to the implementation in [16]). A total of 4096 features $\left({ℱ}_{DL}\right)$ were extracted from each patient, which were finally fed to our LASSO model for survival prognostication, to compute $Risc\left({ℱ}_{DL}\right)$ for every patient. We also conducted a hybrid approach, where we combined ${ℱ}_{DL}$ with ${ℱ}_{rDepTH}$ into the survival model to assess if this combination will improve the performance of ${ℱ}_{rDepTH}$ in survival prognostication.

## V. Results

In order to assess the robustness of r-DepTH features with respect to intra-site variability, our experiments were set up such that the survival models were once trained on each data cohort and tested on the two other cohorts independently. Hence, we report the survival results for 1) training with CCF cohort $\left(S_{t}^{CCF}\right)$ and testing on TCIA $\left(S_{v}^{TCIA}\right)$ and MCW cohorts $\left(S_{v}^{MCW}\right)$, 2) training with TCIA cohort $\left(S_{t}^{TCIA}\right)$ and testing on CCF $\left(S_{v}^{CCF}\right)$ and MCW cohorts $\left(S_{v}^{MCW}\right)$, and 3) training with MCW cohort $\left(S_{t}^{MCW}\right)$ and testing on TCIA $\left(S_{v}^{TCIA}\right)$ and CCF cohorts $\left(S_{v}^{CCF}\right)$.

### A. Survival Risk Assessment Using r-DepTH

LASSO survival analysis while employing $Risc\left({ℱ}_{rDepTH}\right)$ yielded 10 features, listed in supplementary material, when employing each of the 3 cohorts for training ($S_{t}^{TCIA}$, $S_{t}^{CCF}$, and $S_{t}^{MCW}$). Interestingly, there were 5 COLLAGE features that were consistently picked by the regression model across the three training cohorts (e.g., skewness of correlation in enhancing lesion and median of correlation for necrotic core). The KM curves obtained for $S_{v}^{TCIA}$ and $S_{v}^{MCW}$ demonstrated significant differences between the two risk groups (Figure 2.1(e)), with *p*-value = 6.2 × 10^−13^ for $S_{v}^{TCIA}$ and *p*-value = 4 × 10^−9^ for $S_{p}^{MCW}$. Similarly, training the model with TCIA cohort $\left(S_{t}^{TCIA}\right)$ resulted in significant differences between the two risk groups in $S_{v}^{CCF}$ and $S_{v}^{MCW}$ (Figure 2.2(e)), with *p*-values of 0.05 and 9.2 × 10^−5^ for $S_{v}^{CCF}$ and $S_{v}^{MCW}$, respectively. Finally, training the model with MCW cohort $\left(S_{t}^{MCW}\right)$ resulted in significant differences between the two risk groups in $S_{v}^{TCIA}$ and $S_{v}^{CCF}$ (Figure 2.3(e)), with *p*-values of 1.7 × 10^−10^ and 6.5 × 10^−12^ for $S_{v}^{TCIA}$ and $S_{v}^{CCF}$, respectively. Additional measures for all these experiments are provided in Table IV.

**Fig. 3.**
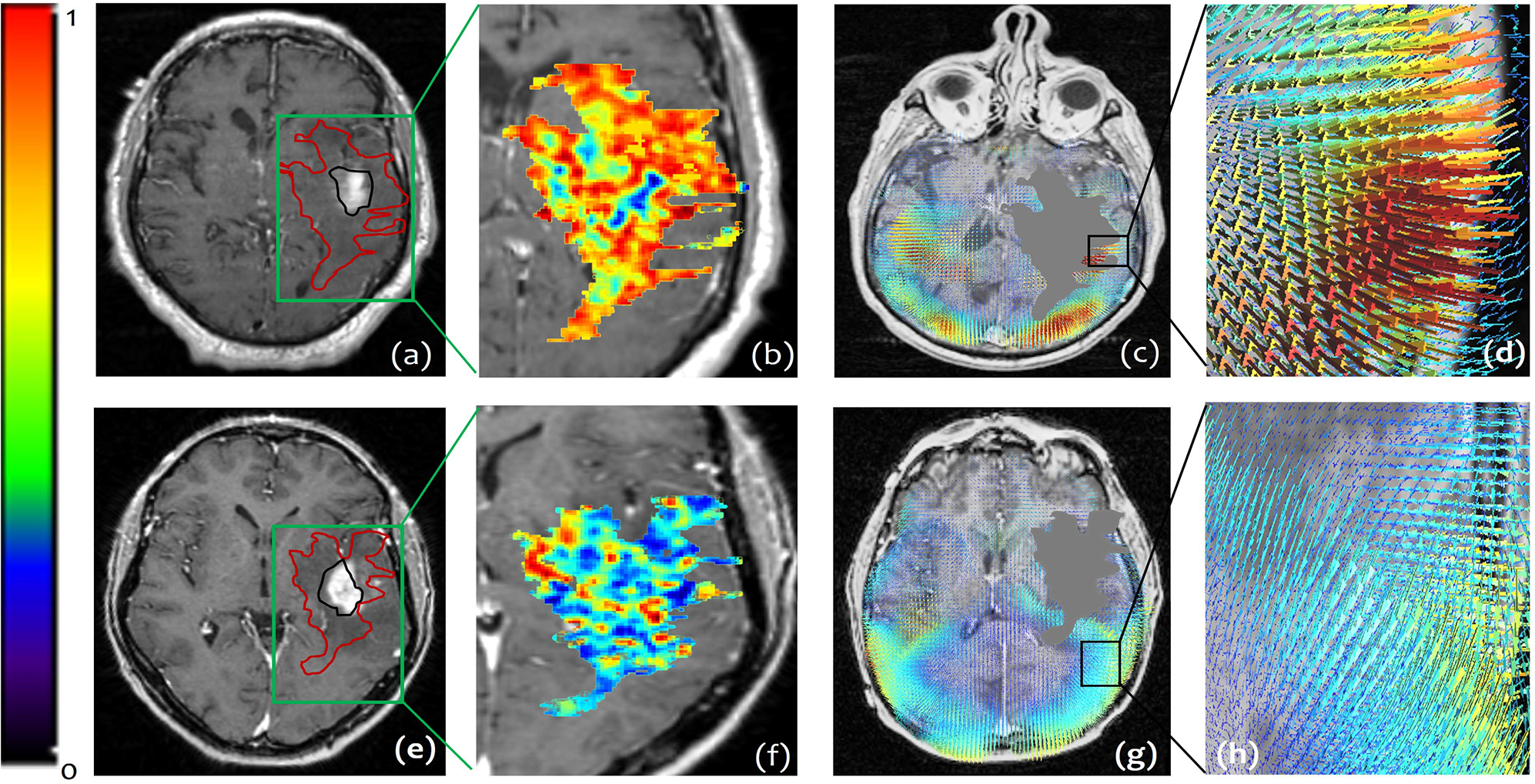
Two subjects from $\left(S_{t}^{CCF}\right)$; a patient with poor survival (top row), OS = 30 days, and a patient with prolonged survival (bottom row), OS = 691 days. (a), (e) show the corresponding Gd-T1w MR scans of the two patients with their tumors segmented into 2 compartments: enhancing lesion (outlined in black) and peri-tumoral area (outlined in red). (b), (f) demonstrate the COLLAGE heatmaps generated for each of the two subjects, with higher values (red) being more prevalent in the patient with poor survival, compared to the patient with prolonged survival. (c), (g) illustrate the extracted deformation field magnitudes respectively for each of the two patients. For the patient with poor survival (d), higher magnitude values (represented in red) were observed in close proximity of the tumor, whereas lower values were observed (blue) for the patient with prolonged survival (h).

Qualitative differences for both COLLAGE and deformation fields for two GBM subjects, one with poor survival (OS = 30 days) (top row) and one with prolonged survival (OS = 691 days) (bottom row), are presented in Figure 3. The patient with poor survival seemed to exhibit higher values of the COLLAGE feature (Kurtosis of Energy) (Figure 3(b)), compared to the patient with prolonged survival (Figure 3(f)). Similarly, the deformation field magnitudes for Skewness (measure of data asymmetry) at 10*mm* are visualized, and seem to reflect higher values for the patient with worse survival (Figure 3(d)), both in close proximity as well as distal to the tumor, compared to that for the patient with improved survival (Figure 3(h)).

### B. Comparative Approaches

#### 1) Risk-Scores Using Clinical Information:

In a uni-variate setting, each of age, gender, and tumor volume did not demonstrate significant differences in the survival groups when training the survival model using $S_{t}^{CCF}$, $S_{t}^{TCIA}$, and $S_{t}^{MCW}$, Table IV. When combining age and gender in a multi-variate setting, significant differences were not observed between the two risk groups across all the cohorts as well, Table IV. Additionally, when combining age and gender information with ${ℱ}_{rDepTH}$ into the survival model to assess if it is going to add value to our descriptor, results did not change when training the model using $S_{t}^{CCF}$. Interestingly, employing this experiment using $S_{t}^{TCIA}$ and $S_{t}^{MCW}$ increased the statistical significance between the two risk groups, Table IV. Additionally, we evaluated molecular markers including MGMT (available for 84 subjects), and IDH (available for 128 subjects), as well as extent of resection (EOR) (available for 120 subjects) for prognosis of GBM survival (the status of the subjects for which the EOR and MGMT were available are provided in the supplementary document). These clinical and molecular parameters unfortunately were not available for MCW and hence could not be evaluated. EOR and MGMT did not demonstrate significant differences between the two risk groups when training the survival model with subjects that had the available information (*p*-value = 0.13, C-index = 0.6 for EOR, *p*-value = 0.3, C-index = 0.52 for MGMT). Similarly, IDH did not demonstrate significant differences in the survival groups when training the model using $S_{t}^{CCF}$ and $S_{t}^{TCIA}$, Table IV. Additionally, in order to control for EOR parameter during the survival analysis, we conducted an experiment where we trained the LASSO survival model with ${ℱ}_{rDepTH}$ using TCIA cohort cases that underwent GTR only (n = 64). The test set for this experiment was the CCF cases that underwent both NTR and GTR (n = 25). We excluded all the other cases with different EOR information (e.g., subtotal resection (STR)) from the analysis to ensure data balance, across the training and test sets. Results showed statistical significance on $S_{t}^{TCIA}$ (*p*-value = 1.5 × 10^−6^, C-index = 0.62), but not on $S_{v}^{CCF}$ (*p*-value = 0.53, C-index = 0.56). Similarly, to control for the methylation status while predicting survival, we conducted an additional experiment, where we trained the survival model with ${ℱ}_{rDepTH}$ using the cases from CCF cohort that were non-methylated (n = 30) and tested on non-methylated TCIA cases (n = 15). Results showed statistical significance on $S_{t}^{CCF}$ (*p*-value = 0.05, C-index = 0.64), but not on $S_{v}^{TCIA}$ (*p*-value = 0.12, C-index = 0.54).

#### 2) Survival Risk-Assessment Using COLLAGE Features From the Tumor and Peri-Tumoral Regions:

When training the LASSO model to obtain $Risc\left({ℱ}_{T,P,N}\right)$ using each of the 3 training cohorts, significant differences were observed when employing $S_{t}^{CCF}$, but not when employing $S_{t}^{TCIA}$ or $S_{t}^{MCW}$. Specifically, the KM curves (Figure 2.1 (c)) obtained from $Risc\left({ℱ}_{T,P,N}\right)$ for $S_{t}^{CCF}$ demonstrated statistically significant differences between the two risk groups for $S_{v}^{TCIA}$ (*p*-value = 1.6 × 10^−4^) and $S_{v}^{MCW}$ (*p*-value = 4.7 × 10^−4^). When employing $S_{t}^{TCIA}$ to obtain $Risc\left({ℱ}_{T,P,N}\right)$, significant differences were not observed between the two risk groups for $S_{v}^{CCF}$ (*p*-value = 0.87) and $S_{v}^{MCW}$ (*p*-value = 0.8), Figure 2.2(c). Similarly, employing $S_{t}^{MCW}$ to obtain $Risc\left({ℱ}_{T,P,N}\right)$ did not result in significant differences between the two risk groups for $S_{v}^{TCIA}$ (*p*-value = 0.4) and $S_{v}^{CCF}$ (*p*-value = 0.14), Figure 2.3(c). Additional measures for all these experiments are listed in Table IV.

#### 3) Survival Risk Assessment Using Deformation Features From the Normal Parenchymal Regions:

When training the LASSO model to obtain $Risc\left({ℱ}_{B}\right)$ using each of the 3 training cohorts, significant differences were observed when employing $S_{t}^{CCF}$, but not when employing $S_{t}^{TCIA}$ or $S_{t}^{MCW}$. The KM curves obtained for $S_{v}^{TCIA}$ and $S_{v}^{MCW}$ when employing $S_{t}^{CCF}$ demonstrated significant differences between the two risk groups (Figure 2.1 (d)), with *p*-values of 0.002 and 0.04 for $S_{v}^{TCIA}$ and $S_{v}^{MCW}$, respectively. Additionally, when training the LASSO model to obtain $Risc\left({ℱ}_{B}\right)$ using $S_{t}^{TCIA}$, significant differences were not observed between the two risk groups for $S_{v}^{CCF}$ (*p*-value = 0.54) and $S_{v}^{MCW}$ (*p*-value = 0.7) (Figure 2.2(d)). Similarly, when training the LASSO model to obtain $Risc\left({ℱ}_{B}\right)$ using $S_{t}^{MCW}$, significant differences were not observed between the two risk groups for $S_{v}^{TCIA}$ (*p*-value = 0.4) and $S_{v}^{CCF}$ (*p*-value = 0.14) (Figure 2.3(d)). Additional measures for these experiments are listed in Table IV.

##### a) Sensitivity analysis on the number of annular bands for deformation features extraction:

As mentioned in Section IV.C, the choice for the size of the annular rings in our experiments (5mm) was based on retrospective studies that have recommended 5mm as a safe clinical target volume margin for GBM tumors [41]. However, for the sake of completeness, in the supplementary document, we provide a comparative analysis that shows the prognostication results using 20 annular bands that were 3mm apart from each other. Results of this experiment suggested that the measures derived from the 5mm band seem to lead to improved metrics (i.e., *p*-values, C-indices), as compared to the 3mm band measurements.

##### b) Extracting deformation features using another registration approach:

We have also compared the performance of the deformation features obtained from ANTs to those obtained using another registration approach, called Greedy [47], to assess whether results obtained from the r-DepTH descriptor would be affected as a function of the registration approach. Our results demonstrated that both registration approaches generated similar measures (p-values and C-indices) suggesting that the r-DepTH descriptor may not specifically rely on a certain registration approach to obtain the deformation heterogeneity features. Results of this experiment are provided in the supplementary document.

**Fig. 4.**
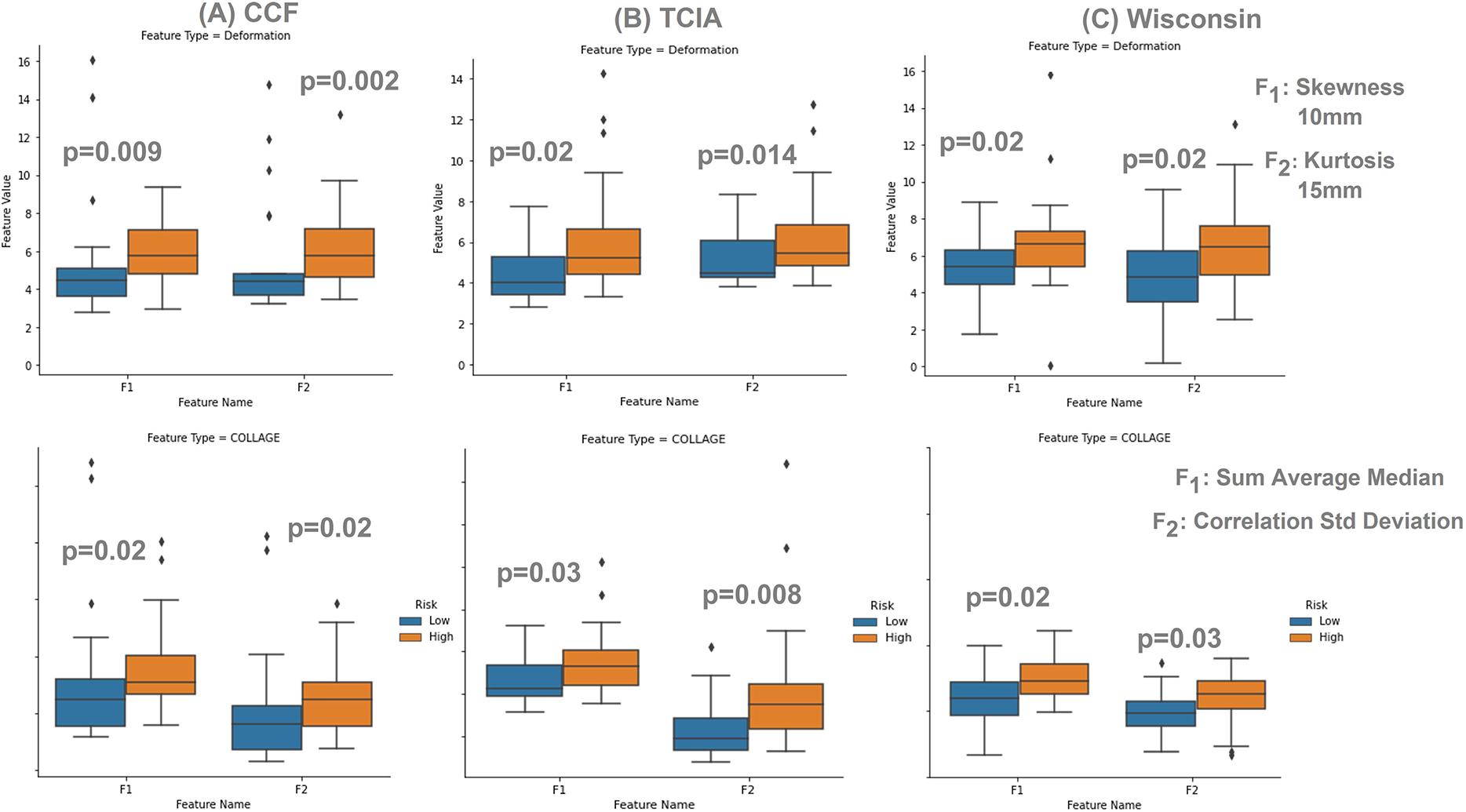
Box plots of four statistically significantly different features between the high-risk and low-risk patients for (A) CCF cohort used for training $\left(S_{t}^{CCF}\right)$, (B) TCIA cohort used for testing $\left(S_{V}^{TCIA}\right)$, and (C) MCW cohort used for testing $\left(S_{V}^{MCW}\right)$. The top row shows 2 deformation features and their p-values for $\left(S_{t}^{CCF}\right)$ (A), $\left(S_{V}^{TCIA}\right)$(B), and $\left(S_{V}^{MCW}\right)$ (C). The first feature is skewness, a measure of data symmetry, at 10 mm and the second one is kurtosis, a measure of the extreme values of a dataset, at 15 mm. The bottom row shows 2 COLLAGE features and their p-values for $\left(S_{t}^{CCF}\right)$ (A), $\left(S_{V}^{TCIA}\right)$(B), and $\left(S_{V}^{MCW}\right)$ (C). The first feature is median of sum average, a measure of the mean of the gray level sum distribution of the image, and the second one is standard deviation of correlation, a measure of the linear dependency of gray levels on those of neighboring voxels. The high-risk group is in orange, whereas the low-risk group is in blue.

Additionally, box-plots for the 2 most discriminative COLLAGE and deformation features on $S_{t}^{CCF}$, $S_{v}^{TCIA}$, and $S_{v}^{MCW}$ are shown in Figure 4. The top deformation features included skewness at 10 mm as well as kurtosis at 15 mm. Similarly, the top 2 COLLAGE features were median of sum average and standard deviation of correlation.

#### 4) Survival Risk Assessment Using Comparative Radiomic and CNN Approaches:

$Risc\left({ℱ}_{Rad}\right)$ obtained from the LASSO model when employing $S_{t}^{CCF}$ did not result in significant differences on $S_{v}^{TCIA}$ (*p*-value = 0.9), or on $S_{v}^{MCW}$ (*p*-value = 0.5), Figure 2.1 (a). Similarly, when training the LASSO model using $S_{t}^{TCIA}$ (Figure 2.2 (a)) and using $S_{t}^{MCW}$ (Figure 2.3(a)), significant differences were not observed between the two risk groups on any of the test cohorts. Table IV details all measures for these experiments.

For the adopted comparative CNN approach, the LASSO analysis to obtain $Risc\left({ℱ}_{DL}\right)$ using $S_{t}^{CCF}$ demonstrated significant differences between the two risk groups for $S_{v}^{TCIA}$, (Figure 2.1 (b)), with *p*-value = 0.015. However, on $S_{v}^{MCW}$, significant differences were not observed between the two groups (Figure 2.1(b)), with *p*-value = 0.4. Similarly, when training the LASSO model using $S_{t}^{TCIA}$, significant differences were not observed between the two risk groups for $S_{v}^{CCF}$ (*p*-value = 0.14) and for $S_{v}^{MCW}$ (*p*-value = 0.2) (Figure 2.2 (b)). Interestingly, training the LASSO model using $S_{t}^{MCW}$ demonstrated significant differences between the two risk groups for $S_{v}^{TCIA}$ (*p*-value = 0.04), but not for $S_{v}^{CCF}$ (*p*-value = 0.64) (Figure 2.3 (b)). Additional measures for these experiments are listed in Table IV.

Additionally, combining ${ℱ}_{DL}$ with ${ℱ}_{rDepTH}$ for survival prognostication did not result in statistically significant differences between the two risk groups across all experiments. Specifically, using $S_{t}^{CCF}$, a *p*-value of 0.38 was obtained on $S_{v}^{TCIA}$ and a *p*-value of 0.62 was obtained on $S_{v}^{MCW}$. Similarly, when using $S_{t}^{TCIA}$, a *p*-value of 0.67 was obtained on $S_{v}^{CCF}$ and a *p*-value of 0.56 was obtained on $S_{v}^{MCW}$. Similarly, *p*-values of 0.75 and 0.86 were obtained for $S_{v}^{TCIA}$ and $S_{v}^{CCF}$ when training the LASSO model using $S_{t}^{MCW}$. The poor results of this hybrid approach could be on account of the poor results obtained from some of the experiments that employed ${ℱ}_{DL}$ alone. Additional measures for these experiments are reported in Table IV.

## VI. Discussion

Highly aggressive tumors such as Glioblastoma (GBM) tend to proliferate way beyond their visible tumor confines on routine MRI scans. For instance, GBM tumors are known to displace the surrounding tissue structures (phenomenon known as mass effect), which often causes herniation in the normal brain around tumor (BAT) parenchymal regions. Additionally, extensive brainstem infiltration in GBM patients which displaces the surrounding structures, is known to lead to worse prognosis in end-stage patients [1], [3], [6]–[8]. In guided-surgery procedures, the additional use of 5-Aminolevulinic acid (5-ALA) has been explored in many studies to assess its impact on overall survival and if it can provide prognostic cues in GBM. For instance, the study in [48] was conducted to assess the impact of additional use of 5-ALA in intraoperative MRI (iMRI) assisted surgery of GBMs on overall survival, and a significant increase of extent of resection (EOR) was found when combining 5-ALA and iMRI compared to use of iMRI alone but could not find any correlations between increase of EOR and progression free survival or overall survival. Also, the study in [49] showed a small but significant increase in survival measures associated with the use of 5-ALA-guided surgical resection with high grade gliomas. Although promising, the use of 5-ALA is still being explored. In the context of routine MR imaging, while previous studies have employed radiomic (textural and shape representations) and deep-learned features obtained from within the visible peri-tumor confines alone [13], [14], [34], no work to our knowledge has explicitly attempted to exploit the quantifiable biomechanical deformation attributes from the BAT regions, as a complementary radiomic feature, in conjunction with features from the tumor and peri-tumoral confines.

In this work, we presented r-DepTH, a radiomic descriptor that leverages both morphological and biomechanical attributes of the tumor regions, and employed it in the context of survival prognostication in GBM. This was achieved by combining measurements that capture subtle tissue deformation features occurring in the surrounding healthy BAT regions due to mass effect, with 3D COLLAGE descriptor, which measures local heterogeneity arising from tumor infiltration, via higher order statistics of local gradient tensors on a voxel-wise basis, from the tumor and peri-tumoral confines. This integrated feature set was then employed to predict overall survival in GBM tumors. The current work builds on the original implementation of r-DepTH [23] and provides substantial improvements in its performance and validation. Our previous work employed a linear discriminant analysis (LDA) classifier to obtain the top features to construct r-DepTH and classify every GBM patient as belonging to poor or improved outcome. In this work, the r-DepTH descriptor is evaluated for prognosticating overall patient survival in GBM tumors using the top features that were obtained using least absolute shrinkage and selection operator (LASSO), along with a cox regression model to stratify patients into low- or high-risk, based on their overall survival (as a continuous variable). We also included textural features from the necrotic core regions existing within the visible tumor confines, in addition to the other two compartments (enhancing lesion and peri-tumoral edema), into r-DepTH descriptor features, for survival prognostication. Further, we conducted extensive comparative strategies to demonstrate improved performance of r-DepTH compared to other approaches in the context of survival prognostication in GBM (radiomic-feature based [46] as well as deep-feature based [16]). Additionally, we performed extensive comparative experiments to assess if the performance of r-DepTH would further improve, by combining r-DepTH features with deep features [16] into the survival model as well as with clinical features such as age and gender. We further evaluated the robustness of the deformation features within r-DepTH across two different registration approaches. Specifically, aside from ANTs registration approach [35] that we used in our experimental design, we evaluated the use of another registration approach, namely, Greedy [47], to obtain the deformations employed within r-DepTH to predict survival. Finally, our evaluation of the r-DepTH descriptor in [23] was limited to a total of *n* = 79 cases; 68 cases for training, and 11 cases for testing. In this work, we evaluated the efficacy of the r-DepTH descriptor on a total of *n* = 207 1.5 Tesla (T) MRI studies (Gadolinium (Gd)-contrast *T*_1*w*_, *T*_2*w*_, *T*_2*w*_-FLAIR), obtained from a multi-institutional cohort (with each cohort used once for training and the other two being used independently for testing (*n*_1_ = 53, *n*_2_ = 75, *n*_3_ = 79)).

Previous radiomic studies have investigated extracting features from the intra-tumor and peri-tumoral boundaries, for survival prediction or improving disease diagnosis. For instance, the study in [21] exploited radiomic shape and texture features extracted from both intra- and perinodular regions (where annular rings were generated around the nodule), to differentiate between cancerous lung nodules and benign masses. Similarly, another study, [22], attempted to prognosticate survival in GBM patients using radiomic texture features extracted from the peritumoral brain parenchyma. The COLLAGE features employed within r-DepTH have previously demonstrated success in characterizing tumor heterogeneity from tumor and peritumoral regions to distinguish similar-appearing pathologies, as well as prognosticate survival, across different applications such as brain, lung, and prostate cancer [34], [50], [51]. In line with our findings, most of these works have reported improvement in their diagnostic/prognostic models with inclusion of both intra and perilesion textural features to characterize disease heterogeneity. Our work builds on these previous findings by combining deformation features from brain around tumor region with the textural features from the lesion and peri-lesional compartments, into our descriptor, to further improve the prognostic model for GBM survival. Results of our work from employing COLLAGE features or deformation features in a siloed manner, to prognosticate survival across different cohorts, demonstrated significant differences between the two risk groups when training the model with one cohort (CCF), but not when training with TCIA or MCW cohorts. Interestingly, combining both feature sets into r-DepTH descriptor yielded statistically significant differences across all training and test cohorts, which demonstrates the impact of the proposed descriptor in predicting survival as well as its generalizability across different sites, through the feature combination that allows for capturing mass effect (via deformation features) as well as tumor infiltration (via COLLAGE features), with both phenomena being associated with more aggressive tumor behavior as well as poor prognosis.

Apart from radiomic features, a few DL approaches in literature have investigated survival prediction in GBM by interrogating features from the visible tumor confines [16]–[18]. When replicating one such model in [16] for GBM survival prognosis on our cohort, the results did not yield significant differences between the high-risk and low-risk groups on some of our experiments (e.g. *p*-value = 0.4, C-index = 0.58 when training with CCF set and testing on MCW cohort). This, we posit, could be on account of training the model on a single slice as compared to the entire 3D volume. Further, the scanner-specific variations in our multi-institutional cohort are likely impacting the results of the adopted CNN approach, similar to previous works demonstrating the same drawback when applying CNN approaches on data collected from multiple institutions [52]. Additionally, when the CNN model was combined with r-DepTH to prognosticate survival, some of our results were not significantly different between the two risk groups, which could be on account of the average performance of the DL approach alone. Also, when combining clinical information, e.g., age and gender, into our descriptor for survival analysis, results showed that this information might aid in improving survival prognostication.

The closest work to our work on exploring structural brain deformations was performed by [53], where structural deformations were obtained from different parcellations in the brain, which were then associated with overall survival in GBM patients. Decreased survival time was found to be associated with increased deformations in certain cognitive and motor-control brain areas. Uniquely, our study found statistically significant structural deformation changes around the tumor boundaries up to 55*mm*, both in the training and the test sets, that contributed to the prognostic signature for distinguishing between high- and low-risk GBM patients. As shown in Figure 4, some of our top deformation features included skewness, an indicator of lack of data symmetry, at 10*mm*. The higher skewness values exhibited by the high-risk group with poor survival (Figure 4) could be linked to the way such aggressive tumors proliferate and exert pressure on BAT, and hence may lead to more lopsidedness in the frequency distribution of the deformation magnitude values at these regions, compared to the group with prolonged survival. Kurtosis, a measure of the extreme values in a dataset, at 15*mm* also turned out to be a top prognostic feature of the two risk groups, where it showed higher values for the high-risk group (Figure 4). This could be on account of the higher heterogeneity of BAT in patients with poor survival, due to active proliferation and herniation beyond tumor confines, leading to higher deformation magnitudes with extreme values.

Our study did have some limitations. One limitation is that we did not explicitly account for direction (i.e phase) attributes of tissue deformations obtained in the BAT region. Further, while r-DepTH in this study was hypothesized to serve as a surrogate measure for the pressure/force exerted by the tumor, this can only be affirmed via controlled in-vivo experiments in a pre-clinical setting. Lastly, molecular and clinical information (i.e IDH, MGMT, EOR) was not available for one of the three cohorts used, and hence could not be used to control for clinical parameters, molecular status, and EOR, while building our prognostic risk assessment models.

## VII. Conclusion

In this work, we presented r-DepTH, an integrated radiomic descriptor that aimed at comprehensively characterizing the field effect from tumor, peri-tumor, and brain around tumor regions, towards predicting overall survival in glioblastoma patients. Our results suggest that combining measurements that quantify subtle biomechanical deformations from the brain around tumor, along with morphological features within the tumor and peri-tumor confines allowed for improved prognostic models for predicting overall survival in GBM, as compared to using clinical variables, as well as using radiomic and deep-learning features from the tumor confines alone. Future work will involve integrating the direction (i.e. phase) attributes of the tissue deformation along with deformation magnitude features to build an integrated prognostic signature of the tumor regions. Additionally, we plan to extend our analysis to a large multi-site retrospective cohort, and eventually to prospectively collected scans, for validation of r-DepTH as a prognostic marker for GBM and other solid tumors.

## Supplementary Material

supp1-3148780

## Figures and Tables

**Fig. 2.**
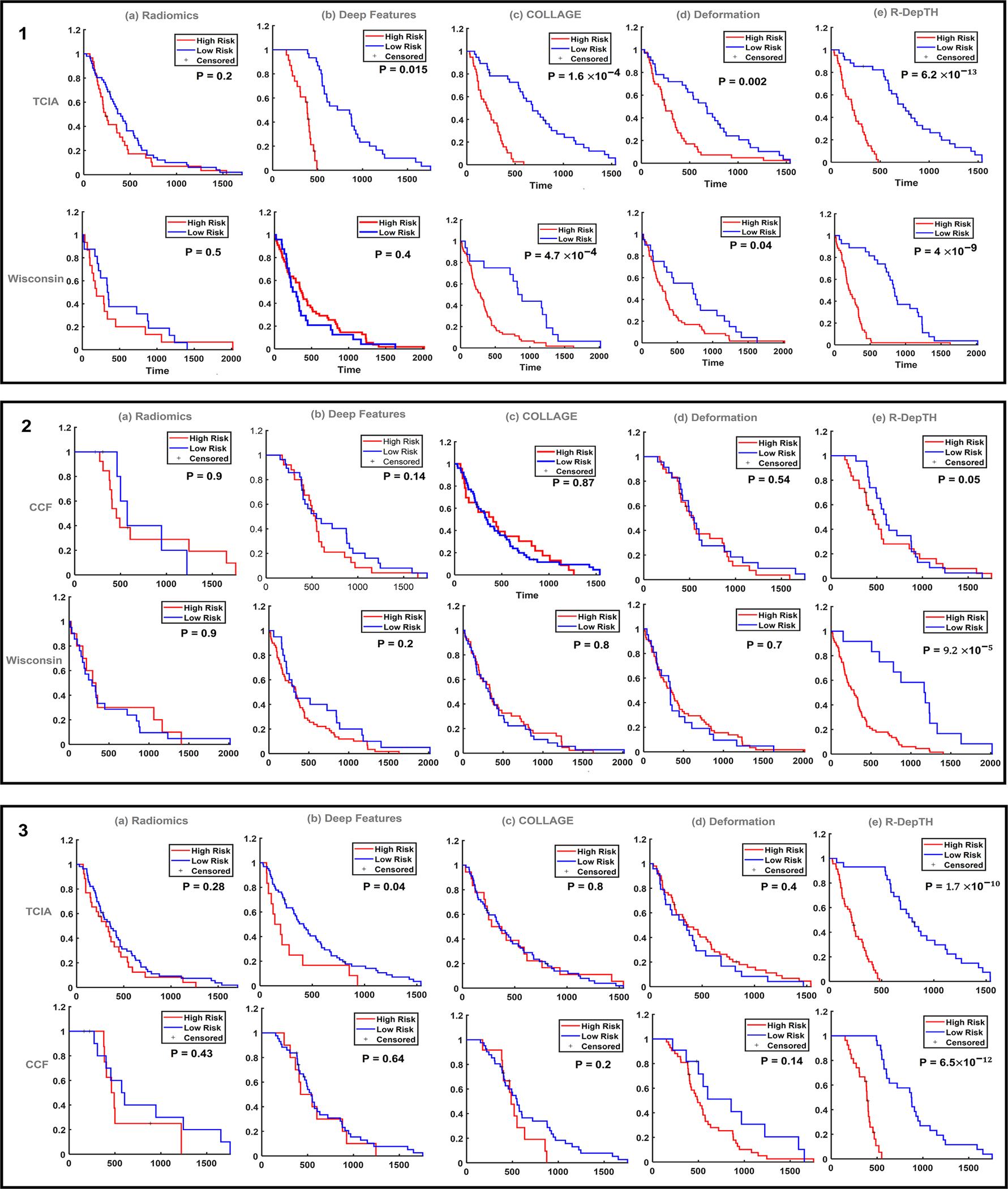
Kaplan Meier curves for estimating overall survival for 1) TCIA $\left(S_{V}^{TCIA}\right)$ and MCW $\left(S_{V}^{MCW}\right)$ cohorts as independent test cohorts when using CCF as the training cohort $\left(S_{t}^{CCF}\right)$, 2) CCF $\left(S_{V}^{CCF}\right)$ and MCW $\left(S_{V}^{MCW}\right)$ as independent test cohorts when using TCIA as the training cohort $\left(S_{t}^{TCIA}\right)$, and 3) TCIA $\left(S_{V}^{TCIA}\right)$ and CCF $\left(S_{V}^{CCF}\right)$ as independent test cohorts when using MCW as the training cohort $\left(S_{t}^{MCW}\right)$. Boxes 1, 2, 3 show the Kaplan Meier curves for estimating survival using (a) the comparative radiomic approach, (b) the DL approach, (c) COLLAGE features, (d) deformation features, and (e) r-DepTH features. X-axis represents the overall survival in days and Y-axis represents the estimated survival function.

**TABLE I:** List of the Notations and Acronyms Used in This Paper

**Notation**	**Description**	**Notation**	**Description**
*I_T_*	intra-tumoral subvolume	*I_p_*	peri-tumoral subvolume
*I_N_*	necrotic subvolume	*I_B_*	normal parenchyma subvolume
*C_T_*	intra-tumoral region voxels	*C_P_*	peri-tumoral region voxels
*C_N_*	necrotic region voxels	$C_{B_{j}}$	normal parenchyma voxels from band *j*
*I_Atlas_*	Healthy brain atlas	*I_mask_*	tumor mask region
${ℱ}_{T}$	COLLAGE features from intra-tumoral region	${ℱ}_{P}$	COLLAGE features from peri-tumoral region
${ℱ}_{N}$	COLLAGE features from necrotic region	${ℱ}_{T,P,N}$	COLLAGE features from tumor compartments
${ℱ}_{B}$	Deformation features	${ℱ}_{rDepTH}$	r-DepTH descriptor features
*θ*(*c*),*ϕ*(*c*)	Principal orientations for COLLAGE	*M* ^*θ*^ *M* ^*ϕ*^	Co-occurrence matrices for COLLAGE
${ℱ}_{rad}$	Comparative radiomic approach features	${ℱ}_{DL}$	Deep features
$S_{t}^{CCF}$	CCF cohort as training set	$S_{v}^{CCF}$	CCF cohort as test set
$S_{t}^{TCIA}$	TCIA cohort as training set	$S_{v}^{TCIA}$	TCIA cohort as test set
$S_{t}^{MCW}$	MCW cohort as training set	$S_{v}^{MCW}$	MCW cohort as test set
**Acronym**	**Description**	**Acronym**	**Description**
TCIA	The Cancer Imaging Archive	CCF	Cleveland Clinic
MCW	Medical College of Wisconsin	*S_t_*	Training set
*s_v_*	Test set	*Risc*	Survival risk score
KM	Kaplan Meier	C-index	Concordance Index
HR	Hazard Ratio	CI	Confidence Interval
EOR	Extent of resection	GTR	Gross total resection
NTR	Near total resection	STR	Subtotal resection

**TABLE II:** Description of Patient Demographics for the Three Cohorts Used in This Study

Group	TCIA	CCF	MCW
Number	75	53	79
Mean of age (Years)	59.6	59.5	61.5
Gender	45 males 30 females	36 males 17 females	39 males 40 females
OS (mean ± STD) (days)	467.4 ± 392.7	520.2 ±376.6	468.7 ±430.8
Censored Subjects	2	3	0
Extent of Resection	*n*=67 Gross total resection: 64 Biopsy: 3	*n*=53 Gross total resection: 16 Near total resection: 9 Neuroblate:7 Biopsy: 6 Subtotal Resection: 15	N/A
MGMT	*n*=38 Methylated: 23 Non-meth: 15	*n*=46 Methylated: 16 Non-meth: 30	N/A
IDH	n = 75 Wild Type: 73 Mutant: 2	n = 53 Wild Type: 47 Mutant: 6	N/A

**TABLE III:** Comparative Strategies to R-Depth

Approach	Extracted Features/ Parameters	Final Features		
		$S_{t}^{CCF}$	$S_{t}^{TCIA}$	$S_{t}^{MCW}$
Radiomic-based	4376 features: maximum, minimum, variance, standard deviation, skewness, kurtosis, mean, and median of: Intensity histogram distributions, Texture, Shape, and Spatial features	18	20	15
CNN-based	4096 features: Weight decay = 5 × 10^−4^ Momentum = 0.9 Initial learning rate = 10^−2^	24	30	26
Clinical	6 features: Uni-, Multi-variate settings of: Age, Gender, Tumor Volume, EOR, MGMT, IDH	-	-	-
COLLAGE	390 features: Mean, Median, Skewness, Standard deviation, Kurtosis of the 13 Haralick statistics for each tumor region	11	11	10
Deformation	60 features: Mean, Median, Skewness, Standard deviation, Kurtosis of the 12 annular bands of the BAT region	10	10	10

**TABLE IV:** *p*-Value, Concordance Index, Hazard Ratio, and 95% Confidence Interval for the Experiments Conducted, on the Independent Test Sets for Each of the 3 Training Cohorts

	Feature	$S_{v}^{CCF}$			$S_{v}^{MCW}$			$S_{v}^{TCIA}$		
		*p*-value	C-index	HR (95% Cl)	*p*-value	C-index	HR (95% Cl)	*p*-value	C-index	HR (95% Cl)
Univariate	Age	0.9, 0.2	0.5, 0.59	1 (0.6 – 2), 1 (0.9 – 2)	0.2, 0.2	0.6, 0.53	1 (0.9 – 2), 1 (0.9 – 2)	0.2, 0.4	0.6, 0.5	1 (0.9 – 2), 1 (0.7 – 2)
	Gender	0.4, 0.42	0.54,0.54	0.7(0.5 – 1), 1 (0.5 – 1)	0.63, 0.7	0.53, 0.5	1 (0.7 – 2),2(0.6 – 0.9)	0.3, 0.25	0.56,0.59	1 (1 – 2), 1 (1 – 2)
	IDH	0.7, –	0.5, –	0.9 (0.9 – 1), –	0.8, –	0.5, –	1 (1 – 3), –	–, –	–, –	–, –
	Tumor Volume	0.12, 0.2	0.61,0.58	2 (2 – 2.4), 1 (1.5 – 2)	0.07, 0.7	0.52,0.46	2 (2 – 3), 0.9 (0.6 – 1)	0.3,0.32	0.56,0.55	2 (1 – 2), 2 (1.5 – 2)
Multivariate	Age, Gender	0.75, 0.2	0.5, 0.59	1 (0.8 – 2), 1 (0.9 – 2)	0.3, 0.23	0.6, 0.55	1 (0.8 – 2), 1 (0.9 – 2)	0.2, 0.3	0.61,0.57	1 (0.9 – 2),2 (1 – 2)
	COLLAGE	0.87, 0.8	0.5, 0.5	1 (0.5 – 2), 1 (0.6 – 2)	1.6 × 10^4^,4.7 × 10^−4^	0.68,0.64	8 (4 – 14), 3 (2 – 4)	0.8, 0.2	0.52,0.59	0.9 (0.5 – 2),2 (0.8 – 4)
	Deformation	0.54, 0.7	0.6, 0.6	1 (0.7 – 2), 1 (0.5 – 2)	0.002, 0.04	0.63,0.6	2(1– 4), 2 (1 – 3)	0.4, 0.14	0.6, 0.57	0.8 (0.5 – 1),2(0.9 – 3)
	Radiomics	0.9, 0.9	0.5, 0.5	1 (0.4 – 3),0.5 (0.4 – 2)	0.2, 0.5	0.6, 0.5	1.4 (0.9 – 2),1(0.7 – 3)	0.28, 0.43	0.65,0.54	1 (0.8 – 2), 2 (0.6 – 6)
	DL	0.14, 0.2	0.6, 0.5	2 (0.6 – 8), 2 (0.8 – 3)	0.015, 0.4	0.63, 0.58	1(0.6 – 1.6),0.8(0.5 – 1)	0.04, 0.64	0.65, 0.6	3 (1 – 6),1 (0.6 – 3)
	**rDepTH**	0.05, 9.2 × 10^−5^	0.6, 0.7	1 (0.7 – 2), 3 (2 – 5)	6.2 × 10^−13^,4 × 10^−9^	0.7, 0.65	10 (6 – 19), 5 (3 – 8)	1.7 × 10^−10^,6.5 × 10^−12^	0.63,0.75	12 (6 – 21),24(10 – 57)
	r-DepTH, Age, Gender	9 × 10^−4^,7 × 10^−6^	0.75, 0.7	3 (2 – 6), 3 (2 – 5)	6.2 × 10^−13^,4 × 10^−9^	0.7, 0.65	10 (6 – 19), 5 (3 – 8)	2 × 10^−11^, 2 × 10^−11^	0.64,0.78	8 (4 – 14), 23 (9 – 55)
	rDepTH + DL	0.67, 0.56	0.6, 0.6	1 (0.6 – 3), 1 (0.5 – 1)	0.38, 0.62	0.61, 0.5	1(0.8 – 2), 0.8(0.4 – 2)	0.75, 0.86	0.6, 0.5	0.9(0.6 – 2), 1(0.6–2)
